# Applying Critical Discourse Analysis to Cross-Cultural Mental Health Recovery Research

**DOI:** 10.2196/64087

**Published:** 2025-02-21

**Authors:** Yasuhiro Kotera, Riddhi Daryanani, Oliver Skipper, Jonathan Simpson, Simran Takhi, Merly McPhilbin, Benjamin-Rose Ingall, Mariam Namasaba, Jessica Jepps, Vanessa Kellermann, Divya Bhandari, Yasutaka Ojio, Amy Ronaldson, Estefania Guerrero, Tesnime Jebara, Claire Henderson, Mike Slade, Sara Vilar-Lluch

**Affiliations:** 1 Institute of Mental Health School of Health Sciences University of Nottingham Nottingham United Kingdom; 2 Center for Infectious Disease Education and Research Osaka University Suita Japan; 3 Health Service and Population Research Department King's College London London United Kingdom; 4 Harvard T.H. Chan School of Public Health Harvard University Boston, MA United States; 5 Department of Community Mental Health & Law National Institute of Mental Health Tokyo Japan; 6 Department of Social Psychology and Quantitative Psychology University of Barcelona Barcelona Spain; 7 Faculty of Nursing and Health Sciences Nord University Namsos Norway; 8 School of English University of Nottingham Nottingham United Kingdom

**Keywords:** critical discourse analysis, cross-cultural mental health recovery research, linguistic analysis, social inequality, mental health, recovery research, language, social inequalities, qualitative analytical approach, linguistic expressions, discourse, analysis, framework, inequalities, CDA

## Abstract

The purpose of this paper is to demonstrate how critical discourse analysis (CDA) frameworks can be used in cross-cultural mental health recovery research. CDA is a qualitative approach that critically appraises how language contributes to producing and reinforcing social inequalities. CDA regards linguistic productions as reflecting, consciously or unconsciously, the narrators’ understandings of, or attitudes about, phenomena. Mental health recovery research aims to identify and address power differentials, making CDA a potentially relevant approach. However, CDA frameworks have not been widely applied to mental health recovery research. We adapted established CDA frameworks to our cross-cultural mental health recovery study. The adapted methodology comprises (1) selecting discourses that indicate positive changes and (2) considering sociocultural practices informed by relevant cultural characteristics identified in our previous research, without placing value judgments. Our adapted framework can support cross-cultural mental health recovery research that uses CDA.

## Critical Discourse Analysis and Mental Health

Critical discourse analysis (CDA) is a qualitative analytical approach that critically appraises how language contributes to the production and reproduction of social inequalities through the examination of authentic uses of language [1,2]. CDA considers that linguistic expressions reflect the speakers’ and writers’ conscious or unconscious perceptions or opinions towards phenomena [1,2]. CDA used in mental health research is grounded in the belief that people’s mental illness experiences are represented in the language they use [3]. This belief underscores the value of analyzing various forms of textual expression, such as first-person narratives and other linguistic representations. CDA can offer profound insights into the personal accounts of mental illness experiences and the societal perceptions surrounding specific mental illnesses.

The application of CDA to mental health has become an important area of research. Informed by corpus linguistics approaches (software-assisted examination of large collections of digitized texts), scholars have delved into media depictions of mental health conditions, as evidenced by Karaminis et al’s [4] examination of autism portrayal in the British press. Likewise, Balfour [5] explored schizophrenia representations in the British press, while Price [6] scrutinized the perpetuation of mental health stereotypes resulting from media portrayals. The critical approach of CDA turns linguistic analysis into an engaged social enterprise; in these studies, it reveals engrained prejudices towards mental health conditions reproduced in mainstream media and how stereotypical representations have evolved over the years.

Moreover, researchers have turned their attention to the rich landscape of digital informal interactions among service users and nonprofessional caregivers. Digital forums and support groups have become significant spaces where individuals share their experiences, exchange information, and provide emotional support related to illnesses. For instance, based on corpus methods, Hunt and Brookes [7] shed light on discussions surrounding anorexia, depression, and diabulimia, while Jones et al [8] appraise the experiences of individuals with psychosis and bipolar disorder. Corpus-informed CDA is actively used to identify patterns of language use in large text-data collections, which deepens understanding of how language shapes our perceptions of mental health and illness.

First-person narratives of mental health experiences are another emerging field. This includes investigations into autobiographical accounts, such as stylistic studies on the experiences of voice hearing narrated by individuals with schizophrenia [9,10]. These stylistic studies have provided refined typologies of voices in auditory hallucinations in schizophrenia, bringing new insights into the phenomenology of the condition. Additionally, CDA studies have evaluated naturally occurring narratives, such as the examination of men’s accounts of depression [11]. CDA research on naturally occurring narratives highlights the importance of lived-experience insights in gaining a more nuanced understanding of living with a mental health condition, both from individual and social perspectives. CDA studies, and discourse studies more generally, have also examined the construction of the self in interaction—eg, Meehan and MacLachlan [12] on schizophrenia, and Kłosińska and Ziółkowska [13] on dementia. Studies on self-identity construction among individuals with dementia, for example, have provided evidence that a sense of self is maintained in the later stages of dementia. This is achieved through the reliance on previous social identities and lexical repertoires to give meaning to the present [13]. A tenet of CDA is that discourse is ideological. In studying narratives of illness and recovery, this may involve examining how dominant discourses around identity (eg, gender, ethnicity) can influence the experience of illness, potentially affecting treatment and recovery (eg, normative ideologies of masculinity may underpin men’s experience of depression [11]).

CDA has been employed extensively within mental health research. Nevertheless, the specific application of CDA within one area of mental health research, namely “mental health recovery research,” remains largely unexplored to date [14]. In this paper, we will present one example of using established CDA frameworks in our cross-cultural mental health recovery study [15].

## Mental Health Recovery

In mental health research, mental health recovery has been receiving attention [16]. Mental health recovery is “a deeply personal, unique process of changing one’s attitudes, values, feelings, goals, skills, and/or roles” and “a way of living a satisfying, hopeful, and contributing life even within the limitations caused by illness” [17]. Approaches oriented towards mental health recovery, known as “recovery-oriented approaches,” have been implemented in mental health services globally, and their positive impacts have been reported, such as empowerment, hope, autonomy, and reduced stigma in service users [18,19], as well as positive attitudes and understanding towards service users in mental health staff [20]. Recovery-oriented approaches are also helpful in understanding the mental health experiences of people with mental disorders such as bipolar disorders [21,22]. Owing to these benefits, mental health recovery has been embedded in many national policies [23-29].

Both CDA and mental health recovery value highlighting and addressing inequalities. CDA’s fundamental principle is to identify inequalities in linguistic and social practices [30]. Similarly, mental health recovery aims to address power differentials in mental health practice and places the person living with a mental health experience at the center [31]. Mental health recovery is deeply rooted in social justice principles, empowering marginalized voices, reducing stigma and discrimination, and promoting equality [32]. This overlap between CDA and mental health recovery research suggests their compatibility, and yet the application of CDA to this field remains relatively unexplored. The current status of psychiatric discourse is diagnostic-driven rather than person-centered, marginalizing service users’ lived experience rather than empowering them, biologically deficit-focused rather than strength-based, and regarding service users as passive patients rather than as individuals with unique needs, goals, and human rights [33]. Research on mental health recovery, empowered by CDA, has the potential to drive cultural changes in the current psychiatric discourse.

## Our Study

Recovery colleges (RCs) are one of the recovery-oriented approaches that are actively used globally [34] and are regarded as a mental health innovation [35]. RCs are learning-based support systems offering information, social support, and skill development for people with mental health symptoms, carers, and staff [36]. RCs are open to anyone interested in mental health and recovery, such as service users, carers, and professionals. People can directly self-enroll in courses offered at an RC. RCs are often free to use (eg, those offered by NHS) or may charge a modest fee (eg, £3 [equivalent to US$ 3.73 as of January 28, 2025] in some RCs in Japan). The duration and length of RC courses vary (eg, a one-off 2-hour session or 5 weekly 3-hour mindfulness sessions). RCs are advertised through various channels such as mental health services, digital platforms (eg, social media), community outreach (eg, flyers), and word of mouth [15,34]. Since the first RC was developed in England in 2009, there have been 221 RCs established in 28 countries within 15 years [34].

Our recent RC study was informed by CDA frameworks to be able to highlight textual emphases in the RC promotional texts in Japan and England and compared them [15]. We explored how RCs are promoted to the public in Japan and England and compared textual and thematic differences from cross-cultural perspectives. The textual emphases found in each country were in line with the cultural characteristics associated with the RC operational model [37], namely collectivism versus individualism and long-term orientation versus short-term orientation [38]. The texts in RCs in Japan emphasized collectivism (eg, “learn together”) and long-term orientation (eg, highlighting the presence of difficulties now), whereas those in RCs in England emphasized Individualism (eg, “self-management”) and short-term orientation (eg, focusing on skill acquisition) [15].

Specific recommendations were made for the RC operational model, which comprises 12 components [37]. The RC operational model was aligned with individualism and short-term orientation [38]. Our findings indicate some of the components may need to change in order to include under-recognized cultural characteristics such as collectivism and long-term orientation. People in Japan see the RC promotional texts that emphasize collectivism and long-term orientation and attend an RC expecting to engage with activities associated with collectivism and long-term orientation. RCs in Japan need to adapt their operations to meet those expectations, which work disadvantageously to achieve some of the components. For example, one of the components—component 3—evaluates whether an RC actively enquires about the student’s individual needs. This activity is more accepted in individualism than in collectivism. In collectivism, actively enquiring about individual needs can be regarded as rude because expressing individual needs may violate group harmony [39]. Therefore, we proposed that component 3 should include collectivism to ensure RCs oriented to this cultural characteristic are included.

## Critical Discourse Analysis in Recovery Research

Our cross-cultural recovery research was informed by established CDA frameworks [1,2,40]. CDA involves textual analysis [1]. In our study, we examined whether the promotional texts of 61 RCs in England and those of all the 13 RCs in Japan reflected any cultural characteristics in their descriptions of recovery and support provided. The analysis was supported by corpus linguistics tools, which involve a computer-assisted examination of digitized texts (eg, [41,42]). In the analysis, we (1) considered the contexts of production and reception [38] and (2) interpreted the results in relation to sociocultural practices (Table 1). We adapted 2 stages of traditional CDA to better fit cross-cultural mental health recovery research: “selection of the discourse” and “consideration of the sociocultural practice” (see Table 1 for the original versions and adaptations).

Since our study considered RCs, our “selection of the discourse” focused on texts highlighting positive changes rather than inequalities (ie, RCs promotional material), as informed by Bartlett’s and Martin’s positive approach [43,44]. Traditionally, CDA studies predominantly focus on highlighting inequalities and discriminatory practices [46]. This approach is not fully applicable to recovery research that aims not only to highlight individuals experiencing difficulty and societal injustice encompassing social, political, and rights aspects around mental health and recovery [47] but also to underscore the positive impacts on people living with mental health symptoms [19]. Many people living with mental health symptoms, and those who work with them, are well aware of inequalities [48]. In addition to recognizing inequalities, they need changes that address inequalities [35]. Therefore, we have employed a positive approach and considered textual productions aimed at promoting inclusive mental health recovery practices (ie, RCs).

The second adaptation was made when considering the relevance of textual findings for sociocultural practice. The “consideration of sociocultural practice” was informed by cross-cultural findings. In our previous study [38], we used Hofstede’s cross-cultural indexes [45] to identify cultural characteristics that were associated with the RC operational model. These cultural characteristics informed how we interpreted the results of the textual analysis, which revealed that different themes were emphasized in RCs in Japan and England. Drawing our interpretation on empirical cross-cultural research also allowed us to reduce potential value judgments. Cross-cultural theories aim to *understand* different cultures, thus seeking to explain cultural differences without placing value judgments on them [49]. In highlighting inequalities reproduced in texts, many CDA studies have critically evaluated those aspects of advantaged groups that perpetuate discrimination. This explicit critical stance has led to criticisms of being biased for formulating value judgments according to a predefined agenda [50,51]. These evaluative assessments, however, can conflict with the aim of cross-cultural theories to understand different cultures. Therefore, our interpretation of textual differences was informed by empirical cross-cultural research [38] and a cross-cultural theory [45] to move away from value judgments.

The implications of adopting these 2 adaptations—“selection of the discourse” and “consideration of the sociocultural practice”—resulted in tangible recommendations for positive changes. Specific changes were proposed in the discussion section of our study paper, such as the suggestion for component 3 to include collectivism as noted above [15].

**Table 1:** Adapted version of the analytical framework for critical discourse analysis [1,2,40].

Stage of analysis	Description	How it is addressed in our work
Preparation (1): select the discourse	Select a discourse according to your research interests. Originally focused on addressing social injustices and inequalities, (critical) discourse analysis is also applied to discourses that promote positive social change [43,44] **Original version** Select a discourse related to injustice or inequality in society.	Selection of recovery colleges (RCs) promotional texts, a relatively new mental health support system that promotes individual empowerment and recovery through learning in community.
Preparation (2): data gathering	Select data sources, consider any ethical implications involved in data gathering, and prepare the data for analysis.	Identification of RCs in England and Japan, and retrieval of relevant descriptions from their information websites, notably focusing on those presenting RCs and recovery to the public. Translation of Japan RCs descriptions into English. Preparation of 2 .TXT files^a^ (Japan RCs and England RCs datasets) to be used with the software Sketch Engine.
Text analysis (micro-level)	According to research interests: Identify the major underlying themes and subthemes. Examine linguistic choices used to represent social actors or events. Examine the stance taken by the author or speaker. Examine whether the text includes references to other texts (intertextuality).	Corpus linguistics-based analysis supported with the software Sketch Engine to retrieve keywords (single and multi-words) and identification of key themes for each dataset. Examination of concordances (keywords in context) guided by the RQs (ie, construal of RCs and recovery in England and Japan RCs promotional texts). Focus on both the portrayal of the RCs and the role attribution to service users.
Discourse practice (meso-level)	Examine the contexts of production and reception of the text. Consider the goal of the text, who has produced it, and the putative audience.	The context of production (RCs as mental health intervention) and the values of producers (RC managerial staff) and putative audience (RC students) have been studied [38]. For the purpose of this study (ie, construal of RC and recovery), the linguistic analysis has not considered characteristics of the textual register (promotional texts of medical services).
Sociocultural practice (macro-level)	Consider relevant sociocultural or historical factors (ie, context of production) that have conditioned the text. Consider whether the text reflects any sociocultural values. **Original version** Examine social relations that control the production of the text; in addition, examine the reciprocal relations (how the texts affect social practices and structures). How do social practices inform the arguments in the text? How does the text in turn influence social practices?	Examination of RCs as a new mental health support system and its main underpinning philosophies. Interpretation of the main themes emerging from the linguistic analysis based on Hofstede's cultural dimensions theory [45] and Kotera et al’s [38] study on the impact of culture on the RC operational model.

^a^TXT files: text files.

## Conclusions

As both CDA and mental health recovery research share the common goal of identifying and addressing inequalities, our adaptation of established CDA frameworks to cross-cultural mental health recovery research represents a crucial step forward. This paper offered valuable insights to researchers seeking to explore recovery in cross-cultural contexts using CDA. We hope our approach will foster a more evidence-based understanding of recovery and reduce inequalities.

## Abbreviations

CDA: critical discourse analysis
RC: recovery college
TXT file: text file
